# Screening of Regulated and Emerging Mycotoxins in Bulk Milk Samples by High-Resolution Mass Spectrometry

**DOI:** 10.3390/foods10092025

**Published:** 2021-08-28

**Authors:** Gabriele Rocchetti, Francesca Ghilardelli, Francesco Masoero, Antonio Gallo

**Affiliations:** 1 Department of Animal Science, Food and Nutrition, Faculty of Agricultural, Food and Environmental Sciences, Università Cattolica del Sacro Cuore, 29122 Piacenza, Italy; francesca.ghilardelli@unicatt.it (F.G.); francesco.masoero@unicatt.it (F.M.); antonio.gallo@unicatt.it (A.G.); 2 Department for Sustainable Food Process, Faculty of Agricultural, Food and Environmental Sciences, Università Cattolica del Sacro Cuore, 29122 Piacenza, Italy

**Keywords:** milk metabolomics, retrospective screening, UHPLC-Orbitrap, multivariate statistics, mycotoxins

## Abstract

In this work, a retrospective screening based on ultra-high-performance liquid chromatography (UHPLC) coupled with high-resolution mass spectrometry (HRMS) based on Orbitrap-Q-Exactive Focus™ was used to check the occurrence of regulated and emerging mycotoxins in bulk milk samples. Milk samples were collected from dairy farms in which corn silage was the main ingredient of the feeding system. The 45 bulk milk samples were previously analyzed for a detailed untargeted metabolomic profiling and classified into five clusters according to the corn silage contamination profile, namely: (1) low levels of *Aspergillus*- and *Penicillium*-mycotoxins; (2) low levels of fumonisins and other *Fusarium*-mycotoxins; (3) high levels of *Aspergillus*-mycotoxins; (4) high levels of non-regulated *Fusarium*-mycotoxins; (5) high levels of fumonisins and their metabolites. Multivariate statistics based on both unsupervised and supervised analyses were used to evaluate the significant fold-change variations of the main groups of mycotoxins detected when comparing milk samples from clusters 3, 4, and 5 (high contamination levels of the corn silages) with cluster 1 and 2 (low contamination levels of the corn silages). Overall, 14 compounds showed a significant prediction ability, with antibiotic Y (VIP score = 2.579), bikaverin (VIP score = 1.975) and fumonisin B2 (VIP score = 1.846) being the best markers. The k-means clustering combined with supervised statistics showed two discriminant groups of milk samples, thus revealing a hierarchically higher impact of the whole feeding system (rather than the only corn silages) together with other factors of variability on the final mycotoxin contamination profile. Among the discriminant metabolites we found some *Fusarium* mycotoxins, together with the tetrapeptide tentoxin (an *Alternaria* toxin), the α-zearalenol (a catabolite of zearalenone), mycophenolic acid and apicidin. These preliminary findings provide new insights into the potential role of UHPLC-HRMS to evaluate the contamination profile and the safety of raw milk to produce hard cheese.

## 1. Introduction

Milk is an important constituent of the human diet in the Western world [1]. In recent decades, world milk production has increased by over 60%, from 522 million tonnes in 1987 to 843 million tonnes in 2018 [1]. As recently reviewed [2,3], the spectrum of milk metabolites can be deeply influenced by several external factors, such as the season, origin, health status, processing, storage, formulation, and feeding systems. 

In this regard, one of the major concerns when considering the feeding system is related to the potential contamination of silages (such as corn silage) by mycotoxins [4,5]. Mycotoxin contamination, especially in milk, has evoked global concerns regarding feed and food safety due to the toxic effects of mycotoxins in both animals and humans [6,7], including carcinogenic, mutagenic, teratogenic, immunotoxic, and estrogenic potential. Mycotoxins are secondary metabolites produced by several fungi and mainly belonging to the *Aspergillus*, *Penicillium*, *Fusarium*, *Alternaria*, and *Claviceps* strains [8]. These genera can produce a wide range of different mycotoxins for which specific regulations have been established in many countries to protect consumers and livestock from their harmful effects [5]. The great ingestion by dairy cows of regulated mycotoxins has been related to the quality of the feeding systems [9]; however, this aspect remains insufficiently investigated. The available data trying to connect the quality of forages and silages (such as corn silage) with other kinds of the so-called emerging mycotoxins (characterized by being neither routinely determined nor legislatively regulated) are very scarce [5,10]. When dairy cows receive a mycotoxin-contaminated feed, these fungal secondary metabolites can be metabolized and potentially transferred to animal-derived food, including milk and dairy products [8]. This aspect might be of great concern, considering the toxic effects previously mentioned.

To date, few analytical methods have been proposed for the simultaneous identification of mycotoxins using high-resolution approaches in milk and dairy products [11]. Regarding the most advanced analytical methods available for this purpose, ultra-high-performance liquid chromatography (UHPLC) has overcome the limitations of conventional HPLC (e.g., lower separation capacity and speed of analysis), improving sensitivity and resolution using packing materials with smaller particle size [12]. Although tandem mass spectrometry (MS/MS) provides adequate quantification and high efficiency for multi-residue analyses, this strategy is sometimes limited for analysis at trace levels in complex matrices. High-resolution mass spectrometry (HRMS) using Orbitrap technology has made it possible to achieve high resolution and good specificity because of the mass accuracy provided by the HRMS detector, combined with traditional information [12,13]. This technique also enables the retrospective analysis of samples, in contrast to MS/MS, by using appropriate and/or ad hoc databases [12,14,15].

Starting from these background conditions, in this work, a high-resolution UHPLC-Orbitrap mass spectrometry approach was used to evaluate the mycotoxins profile of bulk milk samples collected from dairy farms using corn silages as the main ingredients of the total mixed ration (TMR) (i.e., 30.51 ± 5.84% on a dry matter, DM, basis) [16]. The corn silages were classified in five main clusters according to the mycotoxin contamination, as previously reported in Gallo et al. [5]. On the same milk samples, we have previously demonstrated the potential of untargeted metabolomics to evaluate the impact of contaminated corn silages on the most important chemical classes, thus providing evidence that sphingolipids, together with purine and pyrimidine-derived metabolites, are the most affected groups of metabolites [16]. Therefore, the aim of this work was to provide new insights into the ability of a retrospective screening based on HRMS to assess the mycotoxin profile of bulk milk samples, thus potentially evaluating food safety issues as related to both regulated and emerging toxins.

## 2. Materials and Methods

### 2.1. Collection of Milk Samples

Samples of bulk tank milk (500 mL) (*n* = 45) were taken in the period January–June 2018 from dairy farms located in the Po Valley (Italy). These latter farmed Holstein Friesian housed in free-stall barns without pasture access [16]. The herds were characterized by having on average 38.3% ± 1.9 of primiparous on lactating dairy cows and 2.4 ± 0.2 lactations before culling. The dairy cows were milked twice a day (i.e., morning and afternoon milking sessions) and fed a diet based on the large use of corn silage. In particular, the corn silage represented the main ingredient of total mixed ration (TMR) (i.e., 30.51 ± 5.84% on a dry matter basis). The visited dairy herds provided additional information when considering other ingredients characterizing the TMR, namely other small-grain silages (i.e., 10.65 ± 6.80% on dry matter basis) and hay (i.e., 10.26 ± 5.98% on dry matter basis). The lactating dairy cows were fed with the same corn silages, previously analyzed by Gallo et al. [5], from at least four weeks, thus avoiding collecting milk in the period in which corn silage bunkers were changed. Additional information regarding herd composition, milk yield of lactating groups and milk quality, dry matter intake (DMI) as well as TMR formulation characteristics are reported in our previous published works [5,16]. The corn silages were grouped in five clusters according to the mycotoxin contamination profiles, as reported in our previous work [5], namely: cluster 1 (corn silages contaminated by low levels of both *Aspergillus*- and *Penicillium*-produced mycotoxins); cluster 2 (corn silages contaminated by low levels of fumonisins, and other *Fusarium*-produced mycotoxins); cluster 3 (corn silages contaminated by high levels of *Aspergillus*-mycotoxins); cluster 4 (corn silages contaminated by high levels of *Fusarium*-produced mycotoxins); cluster 5 (corn silages contaminated by high levels of fumonisins and their metabolites; number of samples: 3). The collected 45 bulk milk samples were then classified according to the same corn silages grouping, thus obtaining the following five groups: 18 samples (cluster 1), 17 samples (cluster 2), 2 samples (cluster 3), 5 samples (cluster 4), and 3 samples (cluster 5). The sample legend for each cluster can be found in Table S1.

### 2.2. Extraction Step for UHPLC-HRMS Analysis

Milk samples were extracted according to the method previously reported for untargeted screening [16,17,18]. Following a skimming process by centrifugation (4500× *g* for 10 min at 4 °C), the 45 milk samples (*n* = 3) were thoroughly vortex mixed. Afterwards, an aliquot of 2 mL of each sample was added to 14 mL of acetonitrile (LC-MS grade, Sigma-Aldrich, Madison, CA, USA) acidified with 3% formic acid, mixed by vortexing for 2 min and processed with ultrasounds for 5 min. The samples were centrifuged at 12,000× *g* for 15 min at 4 °C to remove large biomolecules (such as proteins). The supernatants were then filtered through 0.22 μm cellulose syringe filters in amber vials until the further untargeted metabolomic screening. 

### 2.3. Screening of Mycotoxins by UHPLC-HRMS Analysis

The UHPLC-HRMS analysis was based on an untargeted metabolomic approach, by using a Q Exactive™ Focus Hybrid Quadrupole-Orbitrap Mass Spectrometer (Thermo Scientific, Waltham, MA, USA) coupled to a Vanquish ultra-high-pressure liquid chromatography (UHPLC) pump and equipped with a HESI-II probe (Thermo Scientific, USA), as previously reported by [5]. The chromatographic separation was achieved under a water-acetonitrile (both LC-MS grade, from Sigma-Aldrich, Milan, Italy) gradient elution (6–94% acetonitrile in 35 min) using 0.1% formic acid as phase modifier, on an Agilent Zorbax Eclipse Plus C18 column (50 × 2.1 mm, 1.8 μm). For the full scan MS analysis, the acquisition was performed using the positive ionization with a mass resolution of 70,000 at *m*/*z* 200. The automatic gain control target (AGC target) and the maximum injection time (IT) were 1e^6^ and 100 ms, respectively. Additionally, randomized injections of pooled quality control (QC) samples were performed in a data-dependent (Top N = 3) MS/MS mode with full scan mass resolution reduced to 17,500 at *m*/*z* 200, with an AGC target value of 1e^5^, maximum IT of 100 ms, and isolation window of 1.0 *m*/*z*, respectively. For the stage of data-dependent MS/MS, the Top N ions were selected for further fragmentation under stepped normalized collisional energy (i.e., 10, 20, 40 eV). The injection volume was 6 μL and the *m*/*z* range for the full scan analyses was 100–1200. Heated electrospray ionization (HESI) parameters were as follows: sheath gas flow 40 arb (arbitrary units), auxiliary gas flow 20 arb, spray voltage 3.5 kV, and capillary temperature 320 °C. Prior to data collection, the mass spectrometer was calibrated using a Pierce™ positive ion calibration solution (Thermo Fisher Scientific, San Jose, CA, USA). To avoid possible bias, the sequence of injections for milk samples was randomized. Additionally, blank samples (i.e., extraction solvent only) were randomly injected through the sequence. 

### 2.4. Data Processing

The collected data (.RAW files) were converted into abf format using the Reifycs Abf Converter and then further processed using the software MS-DIAL (version 4.60) [19]. Automatic peak finding, LOWESS normalization, and annotation via spectral matching against the database Mass Bank of North America were initially carried out. The mass range 100–1200 *m/z* was searched for features with a minimum peak height of 10,000 cps. The MS and MS/MS tolerance for peak centroiding were set to 0.01 and 0.05 Da, respectively. Retention time information was excluded from the calculation of the total score. Accurate mass tolerance for identification was 0.01 Da for MS and 0.05 Da for MS/MS. The identification step was based on mass accuracy, isotopic pattern, and spectral matching. In MS-DIAL, these criteria were used to calculate a total identification score. The total identification score cut-off was >50%, considering the most common HESI+ adducts. Gap filling using peak finder algorithm was performed to fill in missing peaks, considering 5 ppm tolerance for *m/z* values. The software MS-Finder [20] was also used for in-silico fragmentation of the non-annotated mass features, using different available data sources, such as FoodDB, BMDB, PubChem, T3DB (Toxin) and KNApSAcK libraries, thus reaching a level 2 of confidence in annotation [21]. A custom database containing the mycotoxins previously identified in the corn silage samples [5] was also used for a tentative annotation according to the accurate mass and isotopic profile of each compound and exploiting the MS-DIAL software. 

Finally, calibration curves of authentic standards (purity ≥ 97%) of α-zearalenol (CAS number: 36455-72-8), mycophenolic acid (CAS number: 24280-93-1), apicidin (CAS number: 183506-66-3), and tentoxin (CAS number: 28540-82-1) (from Sigma-Aldrich) were injected considering the concentration range: 0.1–100 ng/mL. Data were finally elaborated in the same software to provide semi-quantitative values. In our experimental conditions and according to literature [13,22], the definition of the limit of detection and limit of quantification is not applicable due to the application of high-resolution mass spectrometric method. However, to ensure quantification, a certain degree of confidence is required. Therefore, the limit of quantification for the semi-quantitative analysis was the lowest calibration level used (0.1 ng/mL).

### 2.5. Multivariate Statistical Analysis

The HRMS data were elaborated using the software MetaboAnalyst [23,24]. Briefly, after data normalization, both unsupervised and supervised multivariate statistics were carried out. The unsupervised approach was based on hierarchical cluster analysis (HCA) and k-means clustering approach, while the orthogonal projections to latent structures discriminant analysis (OPLS-DA) was used as supervised tool. Additionally, the OPLS-DA model validation parameters (goodness-of-fit R^2^Y together with goodness-of-prediction Q^2^Y) were inspected, considering a Q^2^Y prediction ability of >0.5 as the acceptability threshold. Thereafter, the OPLS-DA model produced was inspected for outliers and permutation testing (N > 100) was performed to exclude model over-fitting. The importance of each mycotoxin detected for discrimination purposes was then calculated according to the variable selection method VIP (i.e., variables importance in projection), considering as the minimum significant threshold those values higher than 1, also inspecting the S-plot related to the OPLS-DA model built. As the next step, volcano plots were produced for the comparison between contaminated (cluster 3, 4, and 5) vs. control groups (cluster 1 and 2) by coupling fold-change analysis (cut-off value > 1.2) and ANOVA (*p* < 0.05; post-hoc test: Tukey HSD; multiple testing correction: Bonferroni Family-Wise Error Rate). 

## 3. Results and Discussion 

### 3.1. Profiling of Mycotoxins in the Different Milk Samples

**Figure 1:**
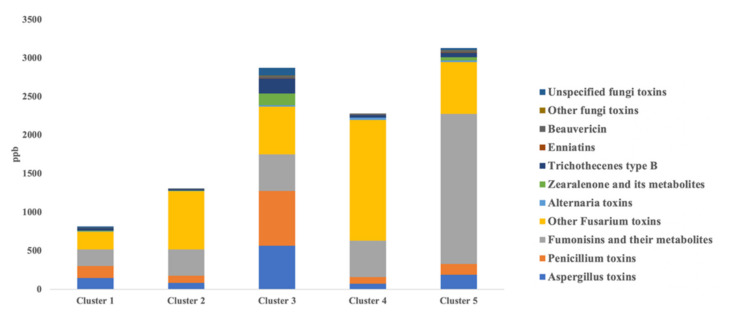
Cumulative values (expressed as µg/kg dry matter) of the major mycotoxins and their metabolites detected in the corn silages belonging to the different clusters.

The starting mycotoxin contamination profile of the different corn silage clusters is summarized in Figure 1, considering each major group of mycotoxins detected, as previously discussed in Gallo et al. [5].

Overall, silages belonging to cluster 1 and cluster 2 were characterized by the lowest contamination levels (cumulatively lower than 1500 µg/kg), while cluster 3, 4, and 5 were highly contaminated by several regulated and emerging mycotoxins (Figure 1). From a qualitative point of view, the corn silages revealed a similar mycotoxin contamination profile, which was mainly associated with *Aspergillus* toxins, *Penicillium* toxins, fumonisins and their metabolites, together with other *Fusarium* toxins. Additionally, our previous work [16] demonstrated that there was a clear impact of contaminated corn silages on the milk metabolomic profiles, and this was particularly true for the metabolism of purines, pyrimidines, sphingolipids, and oxidative stress-related compounds (such as oxidized glutathione). However, it is important to highlight that in this work no information is available on the contamination of the other TMR ingredients potentially affecting the final contamination profile of milk samples. Therefore, after having evaluated separately the fermentative quality characteristics of corn silages [5] and then the untargeted metabolomic profile of bulk milk from dairy cows consuming those contaminated corn silages [16], we exploited a retrospective screening based on high-resolution mass spectrometry to comprehensively investigate the mycotoxin contamination profile of the same bulk milk samples. 

Overall, the retrospective screening following HRMS-based data acquisition allowed us to identify 46 mycotoxins and/or metabolites, which are reported in Table S1 considering their adduct type, reference *m/z*, formula, total identification score (as provided by MS-Dial software), MS1 isotopic spectrum, MS/MS spectrum (where available), and relative abundance values for each sample replicate (*n* = 3). In our experimental conditions, the group composed of other *Fusarium* mycotoxins was found to be the most represented in the final dataset, being composed of 13 compounds (such as fusaric acid and apicidin), followed by 7 *Penicillium* mycotoxins, 5 toxins produced by other fungal strains (including ilicicolin A, ilicicolin B, citreorosein, macrosporin, and iso-rhodoptilometrin) and *Alternaria* mycotoxins (such as alternariol and tentoxin). Additionally, among the 46 mycotoxins detected, 10 compounds were structurally confirmed by means of MS/MS annotations, namely 4Z-infectopyron, kojic acid, fumonisin B2, nivalenol, siccanol, culmorin, 15-hydroxyculmorin, butenolide, beauvericin, and pestalotin (Table S1). Interestingly, the HRMS approach revealed a wide distribution of mycotoxins and some of their metabolites in the bulk milk samples under investigation. In our previous work [5], 69 mycotoxins were identified and quantified in the different corn silages. Therefore, from a qualitative point of view, we found that milk samples were characterized by the 63.7% of mycotoxins found in the corn silages, although this latter was the not exclusive ingredient of the TMR of the visited dairy farms. Future ad hoc studies (such as those based on the evaluation of carry-over phenomena and individual diets for the dairy cows) are extremely necessary to better investigate the toxicological risks for both animals and humans.

### 3.2. Multivariate Analysis on the Different Milk Samples and Discriminant Metabolites

**Figure 2:**
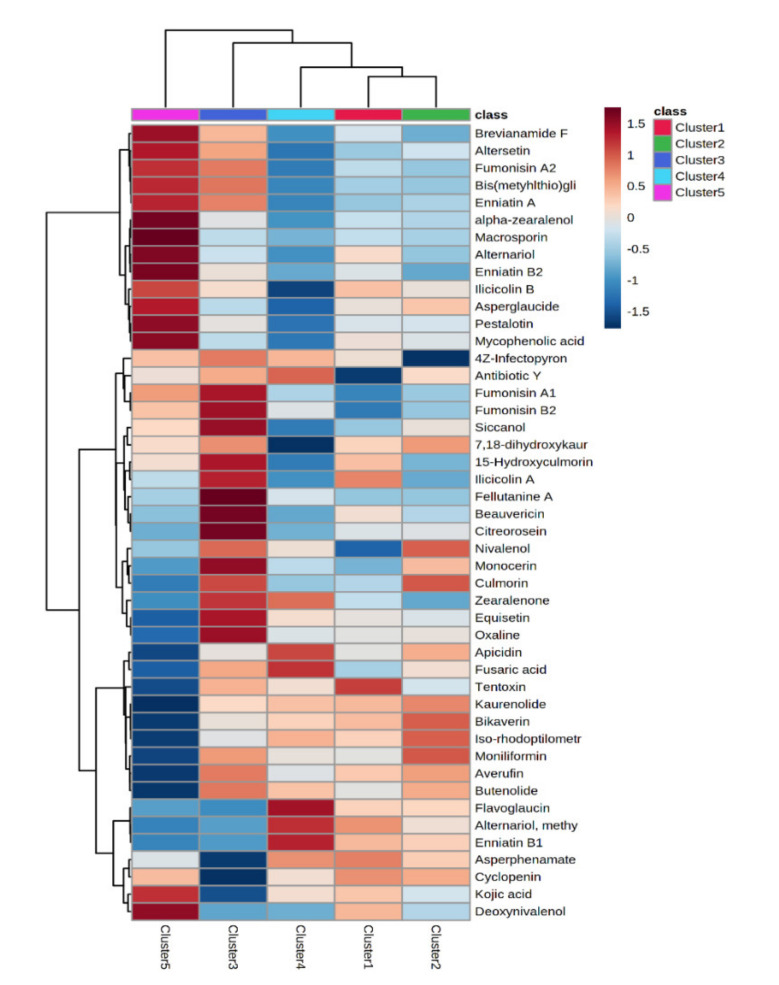
Heat map showing the average clustering (distance measure: Euclidean; clustering algorithm: Ward) of the milk samples according to the mycotoxins detected. The log2 fold-change (FC) values of each abundance were used for the cluster analysis with the Mass Profiler Professional software. Red and blue colors in each column indicate relative up- or down-accumulation of the mycotoxins, respectively.

In this work, a foodomics-based approach was used to retrospectively screen the mycotoxins in the different bulk milk samples to produce hard cheese, to find potential marker compounds of the feeding regimen (typically based on corn silage as the main ingredient). Therefore, starting from the mycotoxin profile reported in Table S1 and previously discussed, we used a multivariate statistical approach based on a hierarchical clustering to naively group the five milk clusters according to the mycotoxins detected. In this regard, the resulting heat map (Figure 2) was built considering the average log fold-change (FC) variations of each mycotoxin detected across the five different contamination clusters. 

As can be observed from the figure, the average mycotoxin distribution based on the corn silage clusters allowed us to group milk samples into two main groups: the first group (on the right side of the heat map) consisted of milk samples belonging to the clusters 1 and 2 (from silages with a low contamination level), cluster 4 (from silages with high levels of non-regulated *Fusarium* mycotoxins), and cluster 3 (high levels of *Aspergillus*-mycotoxins in the silages). Additionally, cluster 5 (i.e., high levels of fumonisins and their metabolites) showed the most differential and exclusive profile. The heat map reported in Figure 2 allowed us to observe an indirect correlation between the contamination profile of corn silages and milk samples, as also reported in our previous work evaluating the global changes of the major chemical classes because of the contaminated feeding systems [16]. Interestingly, cluster 5 was outlined as the most discriminant also in our previous work, thus highlighting the potential of high levels of fumonisins and their metabolites in the corn silages to potentially drive chemical differences in cow milk. Additionally, it was evident from the heat map that some mycotoxins were characterized by both strong up (red color) and down (blue color) accumulation trends in the different milk clusters. 

**Figure 3:**
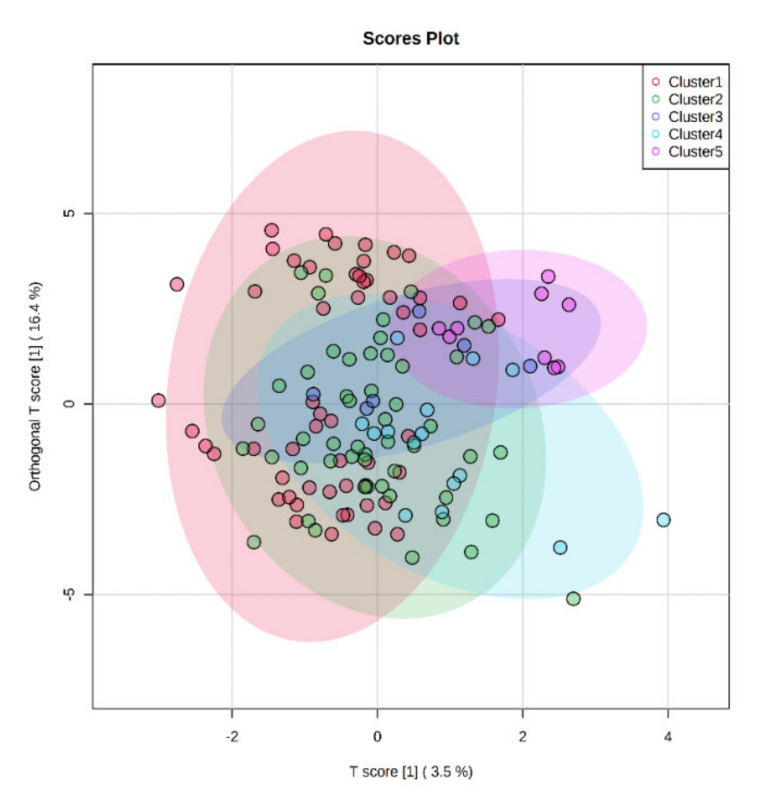
OPLS-DA score plot considering the different milk samples and their cluster, according to the corn silage contamination profile.

The supervised OPLS-DA approach combined with the VIP selection method was then used to extrapolate those mycotoxins characterized by the highest discrimination potential. The OPLS-DA predictive score plot built considering the distribution of the different mycotoxins detected is reported as Figure 3. As can be observed, the 45 milk samples showed a high variability into the score plot space, already observed in the not-averaged unsupervised HCA (not reported), with the orthogonal component explaining the 16.4% of the prediction ability. Additionally, the OPLS-DA model built was characterized by a goodness of prediction lower than 0.5 (i.e., representing the cut-off of acceptability), thus demonstrating a bad correlation between the mycotoxin contamination profile of corn silages and the bulk milk samples, thus resulting in a prediction model not robust enough to be used for discrimination or traceability purposes. This result is likely due to the other ingredients characterizing the TMR. Therefore, further studies are mandatory to better evaluate the final mycotoxin profile detected. As a next step, we extrapolated those mycotoxins better accounting for the differences between the five different milk clusters, using the VIP selection method. Overall, 15 toxins were outlined as the best in terms of discrimination potential (i.e., VIP score > 1), with antibiotic Y (a *Fusarium* mycotoxin) characterized by the best discriminant ability (VIP score = 2.579). Among the VIP markers, the 26.6% consisted of other *Fusarium* mycotoxins, followed by a quite similar numerical distribution (i.e., two toxins per class) for the remaining VIP markers. Among the VIP markers we found also zearalenone (VIP score = 1.358) and its metabolite α-zearalenol (VIP score = 1.098). 

Considering the scarce prediction ability resulting from the supervised OPLS-DA modelling, we decided to combine the information provided by volcano plot (i.e., built by coupling a fold-change analysis with a one-way ANOVA) with the VIP discriminant markers, thus assessing the cumulative LogFC variations of the main classes of mycotoxins. An overview of the different compounds (organized in classes) can be found in Table 1. As a general consideration, the mycotoxins annotated showed differential trends according to the average LogFC values. In this regard, when considering the comparisons with clusters 1 and 2 (considered as a control because of the low contamination levels of corn silages), fumonisins showed an average up-accumulation in milk samples belonging to clusters 3 and 5, whilst *Alternaria* mycotoxins showed no averaged differences, although altersetin was particularly abundant in milk samples characterizing cluster 5. Regarding the group composed of *Aspergillus* mycotoxins, bis(methyl-thio)-gliotoxin was highly and significantly abundant in milk samples belonging to cluster 3 and 5, with average LogFC values of 5.83 and 7.24, respectively. Additionally, regarding zearalenone metabolites, we found a significant down-accumulation of zearalenone in cluster 5 with a corresponding strong and significant (*p* < 0.05) increase in α-zearalenol. Overall, cluster 5 was confirmed again to be the most characteristic in terms of mycotoxin profile; accordingly, it also showed a strong down-accumulation for other *Fusarium* mycotoxins for the comparison with both cluster 1 and cluster 2 (being −1.49 and −1.87, respectively). Regarding the other classes of mycotoxins (such as Enniatins-derived or *Penicillium* toxins), no huge variations were detected for the different milk clusters under investigation (Table 1). 

### 3.3. Discrimination of Milk Samples According to a k-Means Clustering Approach

**Figure 4:**
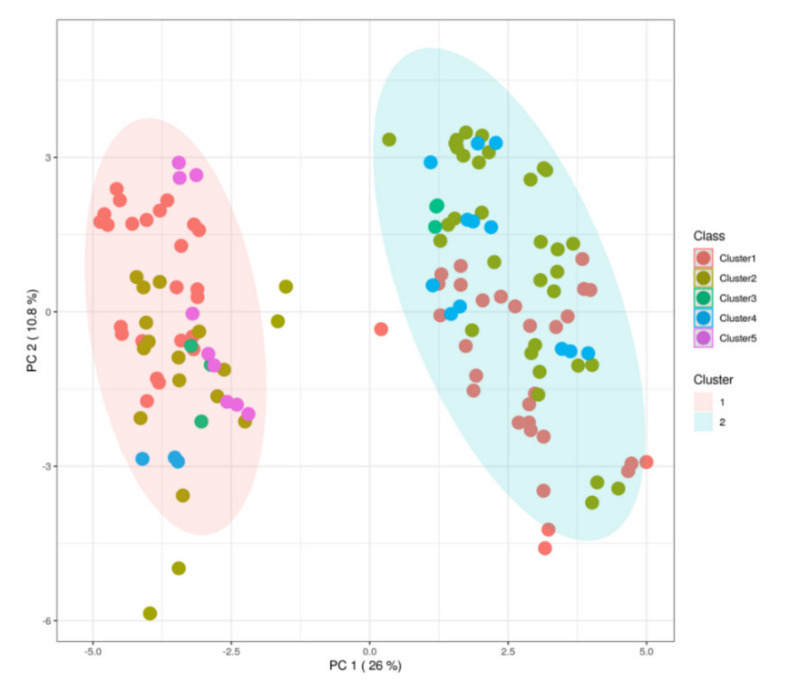
PCA score plot resulting from the unsupervised k-means clustering of the different milk samples under investigation, revealing two major groups according to the mycotoxin contamination profile.

The mycotoxin profile of the different milk samples showed a scarce prediction ability when considering as class discrimination parameter the cluster type used to previously classify the different corn silages [5]. This aspect is not surprising, considering that in our experimental conditions, several variables related to both animals and dairy farm conditions (including the other ingredients of the TMR) may have contributed to the profile observed, not only the contamination of corn silages by mycotoxins. However, the unsupervised statistical approach based on hierarchical clustering dendrogram (Table S1) revealed a tendency in the dataset to discriminate two main groups. This was confirmed by the PCA score plot resulting from the unsupervised k-means clustering approach (Figure 4), with two principal components able to explain the 36.8% of the total variability. Therefore, to find potential marker compounds of the discrimination observed, we used again a supervised statistical approach (OPLS-DA) combined with the S-plot to extrapolate the discriminant features. To this aim, two main groups (Group 1 and Group 2) were considered, and milk samples were assigned to these new groups, accordingly. 

**Figure 5:**
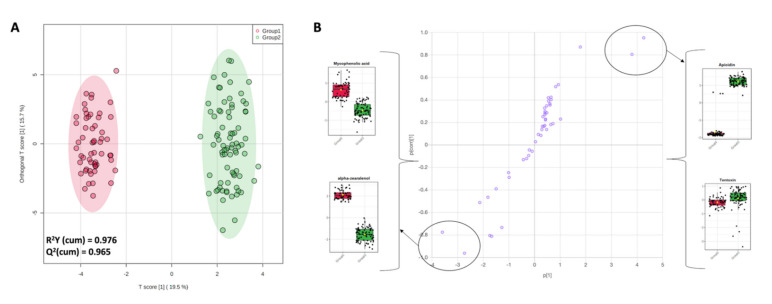
OPLS-DA score plot (**A**) considering the two group of milk samples outlined by the unsupervised k-means clustering approach, together with the S-plot; (**B**) outlining the most discriminant compounds for the comparison Group 1 vs. Group 2, namely mycophenolic acid and α-zearalenol (Group 1) followed by apicidin and tentoxin (Group 2).

Afterwards, a new OPLS-DA prediction model considering the two new groups outlined by the unsupervised k-means clustering was built, and the resulting score plot is provided in Figure 5 (Figure 5A). The new OPLS-DA model was characterized by excellent parameters, recording a goodness-of-fit of 0.976 and a goodness-of-prediction of 0.965, recording a clear separation between the two groups of milk samples. 

Finally, the S-plot related to the OPLS-DA score plot (Figure 5B) was inspected to extrapolate the most discriminant mycotoxins allowing the separation of both principal groups. Interestingly, we found that milk samples belonging to Group 2 were higher in apicidin and tentoxin, while those belonging to Group 1 were mainly discriminated by α-zearalenol and mycophenolic acid (Figure 5B). Considering the clear separation revealed by the OPLS-DA approach and based on the new sample grouping, we aim in a future work to explore more variables able to affect the final mycotoxin profile of milk. In fact, as widely reported in literature [8], various mycotoxins can modify the rumen flora due to their antimicrobial activity. This may decrease the degrading capacity of the rumen, resulting in an unexpected passage rate of intact toxins from other sources. Additionally, changes in the blood–milk barrier due to systemic, and particularly local, infections (mastitis) can affect the integrity of the blood–milk barrier and the pH gradient between blood and milk. Taken together, these effects could, in turn, alter the rate of excretion and facilitate the excretion of mycotoxins that are not expected in milk.

### 3.4. Semi-Quantitative Analysis by UHPLC-HRMS of the Discriminant Markers

As a final step, a semi-quantification in HRMS-mode using authentic standards was carried out considering some of the discriminant marker compounds revealed by the different statistical approaches, namely α-zearalenol, mycophenolic acid, apicidin, and tentoxin. The results obtained are reported in Table 2, as average contents (*n* = 3) for each milk sample. 

As shown in the Table 2, we found a concentration range for the tentoxin of 0.19 to 2.9 ng/mL, with no significant differences between the different milk groups considered according to the k-means unsupervised clustering (i.e., Group 1 and Group 2). Regarding the distribution of tentoxin in the corn silages, Gallo et al. [5] reported a maximum content of 88.9 µg/kg for corn silage samples belonging to cluster 4, followed by cluster 2 (46.2 µg/kg) and cluster 5 (34.3 µg/kg). *Alternaria* species can produce more than 70 toxins, which play important roles in fungal pathogenicity and food safety, since some of them are harmful to humans and animals [25]. The studied *Alternaria* secondary metabolites belong to diverse chemical groups such as nitrogen-containing compounds (amide, cyclopeptides, etc.), steroids, terpenoids, pyranones, quinines, and phenolics. The major *Alternaria* toxins belong to the chemical groups dibenzo-pyrones, which include alternariol and alternariol monomethyl ether and cyclic tetrapeptides represented by tentoxin. These mycotoxins were the most studied metabolites produced by *Alternaria* strains on different substrates (tomato, wheat, blueberries, walnuts, etc.) and some of the main *Alternaria* compounds thought to pose a risk to human and animal health because of their known toxicity and their frequent presence as natural contaminants in food [26]. The cyclic tetrapeptide tentoxin is one of the major *Alternaria* toxins produced, along with dihydrotentoxin and isotentoxin. Their structures differ at the unsaturated bond of the N-methyldehydrophenylalanine moiety, which is hydrogenated into a single bond in dihydrotentoxin, and E configured in isotentoxin. All three compounds are phytotoxins, with tentoxin being the most potent, inhibiting photophosphorylation and inducing chlorosis [27]. However, no toxicological data are available for mammals, and the data on the occurrence of this toxin in food and feed are limited as well. Additionally, according to the scientific opinion provided by EFSA in 2016 [26], the levels of tentoxin in the different food categories considered were the lowest among the four *Alternaria* toxins covered. In this regard, the highest levels were found in samples of sunflower seeds (on average 80 µg/kg). Additionally, although based on limited data, vegetarians seemed to have higher dietary exposure to tentoxin than the general population. Overall, few data are available in literature about absolute quantification or screening of *Alternaria* toxins, such as tentoxin, in milk and dairy products. In a previous work, Izzo and co-authors [12] tentatively identified tentoxin in seven milk samples by using a high-resolution retrospective screening, pointing out the necessity of evaluating other fungal toxic metabolites in milk monitoring studies besides the regulated mycotoxins and their known metabolites. 

Among the VIP markers of the OPLS-DA model we found also zearalenone (VIP score = 1.358) and its metabolite α-zearalenol (VIP score = 1.098). As reported in Gallo et al. [5], the corn silages belonging to cluster 1 were characterized by a total content of zearalenone and derivatives of 95.3 µg/kg, with a great abundance of zearalenone-sulfone (i.e., 91.2 µg/kg). On the other hand, the lowest contents for zearalenone derivatives were reported for cluster 2 (22 µg/kg) and cluster 4 (2.5 µg/kg), whilst the highest values were reported for cluster 3 (i.e., 152.8 µg/kg) and cluster 5 (i.e., 286.6 µg/kg). Accordingly, the highest abundance of zearalenone-sulfone was found in cluster 5, being 284.9 µg/kg. This might be of great concern considering the toxic effects of this compound. Zearalenone and its derivative, α-zearalenol, are a family of phenolic compounds produced by several species of *Fusarium* (such as *F. graminearum, F. culmorum, F. crookwellense, F. sambucinum* and *F. equiseti*), which can infect many important crops such as corn, wheat, sorghum, barley, oats, sesame seed, hay, and corn silage [28]. These fungal toxins have been associated with hyperestrogenism and other reproductive disorders in swine. In sows, a series of reproductive disorders may occur at greater levels of zearalenone in feed (50–100 mg/g feed), including the induction of vulvovaginitis, vaginal and rectal prolapses, delayed onset of the first estrus, infertility characterized by continuous estrus, pseudo-pregnancy, ovarian abnormalities, and pregnancy loss [29]. Additionally, the α-zearalenol metabolite is reported to be three/four times more estrogenic than zearalenone [30]. In our experimental conditions, we found not quantifiable values up to more than 5 ng/mL for α-zearalenol. Interestingly, milk samples belonging to cluster 5 showed the higher average value, according to the highest values of zearalenone derivatives recorded in the silages belonging to cluster 5 (i.e., 286.6 µg/kg). Besides, α-zearalenol was a specific marker of Group 1 of the OPLS-DA model reported in Figure 5, while Group 2 showed no detectable levels (Table 2).

Finally, mycophenolic acid (a *Penicillium* mycotoxin) and apicidin (a *Fusarium* mycotoxin) were found to be two of the most discriminant mycotoxins of the new OPLS-DA model built (Figure 5). To date, no comprehensive information concerning the carry-over of mycophenolic acid from feed to milk is available, although carry-over is possible [31]. However, in our experimental conditions, we found semi-quantitative values of <0.1 up to 1.38 ng/mL in milk samples for this *Penicillium* mycotoxin. This compound presents low acute cytotoxicity on the human intestinal cell line Caco-2 compared to other mycotoxins (such as T-2 toxin, gliotoxin, deoxynivalenol, and patulin) after 48 h exposure; however, it has been shown to possess immunosuppressive effects [31]. According to literature, mycophenolic acid is found mainly in blue-veined cheeses. In fact, Fontaine et al. [31] reported that the 75% of cheese samples analyzed contained a maximum of 705 μg/kg of mycophenolic acid. However, cheeses may be directly contaminated by mycotoxins, such as mycophenolic acid, because of accidental or intentional mycotoxigenic fungal development on the cheese surface or in their core, while the presence of mycophenolic acid in bulk milk is only attributable to potential carry-over from the feed. Therefore, further studies based on strong and targeted monitoring plans of mycophenolic acid in both the TMR and milk (considering individual animals and single diets) are mandatory. As revealed by the semi-quantification of mycophenolic acid reported in Table 2, this compound was a specific marker of the Group 1. Regarding apicidin, no information in the literature is available about its presence in milk and dairy products, and this was also due to its nature of “emerging” mycotoxin. Overall, apicidin (a cyclic tetrapeptide) is a fungal metabolite that exhibits potent, broad-spectrum, antiprotozoal activity and inhibits histone deacetylase activity at nanomolar concentrations [32]. As reported in Table 2, this compound was a specific marker of the Group 2. Apicidin and mycophenolic acid (as emerging mycotoxins) have been identified also in the corn silages previously analyzed by Gallo et al. [5]; however, in our experimental conditions it is impossible to provide carry-over evaluations and further work is mandatory to better evaluate the impact and incidence of the contamination profile.

## 4. Conclusions

In this work, we have assessed the potential of a metabolomics-based approach coupled with a retrospective screening by high-resolution mass spectrometry to evaluate the mycotoxin profile (in terms of both regulated and emerging mycotoxins) of bulk milk samples. Overall, the HRMS approach allowed us to identify 46 mycotoxins, but the accuracy of the prediction was not robust enough and further ad hoc and targeted studies (based on dedicated and optimized extraction conditions and tandem MS analyses) are required to confirm the robustness of the markers proposed. Additionally, by coupling unsupervised (different clustering algorithms) and supervised (such as OPLS-DA) approaches we found the most discriminant mycotoxins driving the separations of the bulk milk samples under investigation. In this regard, among the most discriminant markers we found α-zearalenol, mycophenolic acid, tentoxin, and apicidin. The preliminary semi-quantitative results obtained in this work suggest potential carry-over and metabolization phenomena in milk of the selected mycotoxins, although further ad hoc confirmation studies are mandatory, mainly when considering the toxicity reported in literature for some of the markers identified. 

## Figures and Tables

**Table 1:** Mycotoxins detected by HRMS in the different milk samples. Each compound is provided with its VIP score (from OPLS-DA), log2 fold-change (FC) value and p-value (FWER) resulting from the volcano plot analysis.

Class	Compound	*p*-Value (FWER)	VIP Score (OPLS-DA)	LogFC Cluster 3 vs. Cluster 1	LogFC Cluster 3 vs. Cluster 2	LogFC Cluster 4 vs. Cluster 1	LogFC Cluster 4 vs. Cluster 2	LogFC Cluster 5 vs. Cluster 1	LogFC Cluster 5 vs. Cluster 2
*Alternaria* mycotoxins	Altersetin	*p* > 0.05	<1	2.151	1.562	−1.466	−2.056	3.343	2.753
	Alternariol	*p* > 0.05	<1	−0.064	0.113	−0.341	−0.163	−0.097	0.080
	Alternariol, methyl-ether	0.0011	<1	−0.810	−0.433	0.139	0.516	−1.273	−0.897
	Tentoxin	0.0495	1.119	−0.158	0.272	−0.492	−0.061	−1.258	−0.827
	4Z-Infectopyron	0.0354	<1	0.298	1.072	0.002	0.774	−0.210	0.562
*Aspergillus* mycotoxins	Brevianamide F	0.0065	1.287	0.307	0.671	−0.584	−0.220	0.501	0.865
	Kojic acid	*p* > 0.05	<1	−0.542	−0.513	−0.250	−0.222	−0.267	−0.239
	Averufin	*p* > 0.05	<1	0.531	0.129	0.080	−0.321	−0.640	−1.042
	Bis(methylthio)gliotoxin	0.0066	1.102	5.414	6.242	−2.603	−1.775	6.830	7.657
	Asperphenamate	*p* > 0.05	<1	−5.604	−4.700	−1.351	−0.447	−2.126	−1.222
Fumonisins mycotoxins	Fumonisin A1	0.0494	1.667	2.069	1.671	0.412	0.014	1.084	0.687
	Fumonisin A2	0.0024	<1	3.285	4.041	−4.203	−3.447	4.114	4.870
	Fumonisin B2	0.0294	1.846	2.444	1.918	0.858	0.332	1.096	0.570
Zearalenone and metabolites	Zearalenone	0.0176	1.358	0.420	0.632	0.175	0.386	−0.543	−0.332
	α-Zearalenol	0.0378	1.098	0.416	0.666	−1.264	−1.014	2.822	3.072
Trichothecenes	Deoxynivalenol	*p* > 0.05	<1	−0.396	−0.080	−0.530	−0.214	0.00072	0.316
	Nivalenol	*p* > 0.05	1.114	0.198	0.053	−0.029	−0.174	−0.265	−0.410
Other *Fusarium* mycotoxins	Siccanol	*p* > 0.05	<1	0.335	0.303	−0.261	−0.293	−0.214	−0.246
	Monocerin	*p* > 0.05	<1	0.460	0.287	−0.072	−0.246	−0.376	−0.550
	Moniliformin	5.2 × 10^−6^	1.012	0.442	−0.187	−0.119	−0.748	−1.359	−1.988
	Equisetin	*p* > 0.05	<1	1.166	2.234	−0.060	1.006	−1.493	−0.426
	Culmorin	*p* > 0.05	<1	0.243	0.071	−0.185	−0.357	−0.467	−0.639
	15-Hydroxyculmorin	*p* > 0.05	<1	0.137	0.352	−0.377	−0.161	−0.376	−0.161
	Butenolide	*p* > 0.05	<1	0.116	0.097	−0.099	−0.118	−0.560	−0.578
	Bikaverin	3.4 × 10^−4^	1.975	−1.630	−3.375	−0.734	−2.478	−9.655	−11.400
	Apicidin	*p* > 0.05	<1	0.104	−1.462	3.163	1.596	−4.700	−6.268
	Antibiotic Y	0.0065	2.579	3.395	1.069	0.771	−1.554	2.315	−0.010
	Kaurenolide	0.0024	1.581	−0.178	−0.334	−0.198	−0.354	−1.845	−2.001
	7,1-Dihydroxykaurenolide	*p* > 0.05	<1	0.954	1.572	−1.999	−1.381	−0.011	0.606
	Fusaric acid	*p* > 0.05	<1	0.250	0.175	0.264	0.189	−0.578	−0.653
Enniatins-Beauvericin toxins	Enniatin A	*p* > 0.05	<1	0.647	0.640	−0.418	−0.425	0.564	0.557
	Enniatin B1	*p* > 0.05	<1	−1.421	−0.724	0.767	1.464	−1.944	−1.248
	Enniatin B2	0.0434	1.039	0.058	0.436	−0.466	−0.088	0.458	0.836
	Beauvericin	0.0024	<1	0.438	0.656	−0.429	−0.211	−0.586	−0.368
*Penicillium* mycotoxins	Asperglaucide	*p* > 0.05	<1	−0.452	−0.802	−1.913	−2.263	1.347	0.997
	Pestalotin	*p* > 0.05	<1	0.088	0.167	−1.029	−0.951	0.981	1.0595
	Oxaline	*p* > 0.05	<1	0.428	0.475	−0.174	−0.127	−0.699	−0.652
	Flavoglaucin	0.0111	<1	−1.088	−0.992	0.842	0.938	−1.288	−1.192
	Cyclopenin	*p* > 0.05	<1	−0.515	−0.420	−0.281	−0.187	−0.393	−0.299
	Fellutanine A	*p* > 0.05	<1	1.067	1.129	0.037	0.099	−0.283	−0.221
	Mycophenolic acid	0.0147	<1	−0.312	−0.094	−1.394	−1.175	1.296	1.515
Other fungal metabolites	Ilicicolin A	0.0146	1.706	2.421	6.086	−4.303	−0.639	−2.547	1.117
	Ilicicolin B	0.0467	<1	−0.086	0.734	−3.767	−2.946	−1.029	−0.208
	Citreorosein	0.0369	<1	5.858	5.831	−2.225	−2.252	−2.510	−2.538
	Macrosporin	1.6 × 10^−13^	<1	−4.451	−4.295	−0.415	−0.260	−4.296	−4.147
	Iso-Rhodoptilometrin	3.2 × 10^−5^	1.254	−0.162	−0.462	−0.042	−0.341	−1.301	−1.599

**Table 2:** Semiquantitative analysis based on HRMS (UHPLC-Orbitrap-MS) of tentoxin, α-zearalenol, mycophenolic acid, and apicidin in the different milk samples according to the main groups highlighted by the k-means clustering approach. nd = not detected.

Group (k-Means)	Milk Sample	Tentoxin (ng/mL)	α-Zearalenol (ng/mL)	Mycophenolic Acid (ng/mL)	Apicidin (ng/mL)
Group 1 (left side)	Sample 2	0.86	2.44	1.27	nd
	Sample 6	1.68	3.81	1.08	nd
	Sample 11	1.71	2.91	0.95	nd
	Sample 14	1.03	2.31	0.31	nd
	Sample 16	0.64	1.79	0.52	nd
	Sample 20	2.21	3.06	1.19	nd
	Sample 43	1.08	2.33	0.35	nd
	Sample 45	2.04	3.35	0.34	nd
	Sample 10	1.69	4.94	0.48	nd
	Sample 13	1.93	2.79	0.49	nd
	Sample 21	1.18	5.25	2.68	nd
	Sample 23	0.67	1.06	0.46	nd
	Sample 26	0.71	2.13	0.50	nd
	Sample 32	1.16	2.90	0.79	nd
	Sample 5	1.07	2.90	0.30	nd
	Sample 27	1.07	1.96	0.62	nd
	Sample 4	0.76	1.75	0.28	nd
	Sample 28	1.22	4.93	1.38	<0.1
	Sample 41	0.87	1.80	0.99	nd
Group 2 (right side)	Sample 19	1.01	<0.1	<0.1	0.33
	Sample 22	1.16	<0.1	<0.1	0.23
	Sample 25	2.35	<0.1	0.19	0.28
	Sample 29	1.85	<0.1	<0.1	0.15
	Sample 33	1.05	<0.1	0.16	0.11
	Sample 36	1.79	<0.1	<0.1	0.57
	Sample 37	2.07	<0.1	<0.1	0.27
	Sample 38	2.35	<0.1	<0.1	0.24
	Sample 39	1.16	<0.1	0.15	0.32
	Sample 40	2.06	<0.1	<0.1	<0.1
	Sample 1	0.29	<0.1	<0.1	0.13
	Sample 3	0.19	<0.1	<0.1	0.24
	Sample 7	1.49	<0.1	<0.1	0.20
	Sample 15	1.15	<0.1	<0.1	0.44
	Sample 17	1.28	<0.1	<0.1	<0.1
	Sample 24	1.32	<0.1	<0.1	0.43
	Sample 30	1.71	<0.1	<0.1	0.18
	Sample 31	2.42	<0.1	0.15	0.41
	Sample 34	2.02	<0.1	<0.1	0.55
	Sample 42	2.23	<0.1	<0.1	0.14
	Sample 44	2.88	<0.1	<0.1	0.41
	Sample 8	1.43	<0.1	<0.1	0.71
	Sample 9	<0.1	<0.1	<0.1	0.30
	Sample 12	1.01	<0.1	<0.1	0.15
	Sample 18	2.06	<0.1	<0.1	0.12
	Sample 46	1.46	<0.1	<0.1	0.25

## Data Availability

Not applicable.

## Supplementary Materials

The following are available online at https://www.mdpi.com/article/10.3390/foods10092025/s1, Table S1: dataset resulting from the screening by HRMS of the different mycotoxins annotated in the milk samples (the MS1 and MS/MS isotopic profiles for each compound are provided with both isotopic mass and corresponding relative intensity values) together with the sample legend and the unsupervised grouping (tree plot) of the different milk samples according to the hierarchical clustering approach (built considering the Euclidean similarity measure and ‘Wards’ as linkage rule).
